# Autophagy Is Suppressed in Peripheral Blood Mononuclear Cells During Chronic Obstructive Pulmonary Disease

**DOI:** 10.3390/ijms27125337

**Published:** 2026-06-13

**Authors:** James M. Cooper, Shiye Chen, Susan E. Lester, Julia Kim, Jason Gummow, Thomas Crowhurst, Emily Lawton, Arash Badiei, Phan T. Nguyen, Paul N. Reynolds, Hubertus P. A. Jersmann, Eugene Roscioli

**Affiliations:** 1 Adelaide Medical School, University of Adelaide, Adelaide, SA 5000, Australia; jamesmcoops@gmail.com (J.M.C.); shiye.chen@adelaide.edu.au (S.C.); susan.lester@sa.gov.au (S.E.L.); jason.gummow@adelaide.edu.au (J.G.); thomas.crowhurst@sa.gov.au (T.C.); emily.lawton@sa.gov.au (E.L.); arash.badiei@sa.gov.au (A.B.); phantien.nguyen@sa.gov.au (P.T.N.); paul.reynolds@adelaide.edu.au (P.N.R.); hubertus.jersmann@sa.gov.au (H.P.A.J.); 2 Department of Rheumatology, The Queen Elizabeth Hospital, Adelaide, SA 5011, Australia; 3 Department of Thoracic Medicine, Royal Adelaide Hospital, Adelaide, SA 5000, Australia; julia.kim@sa.gov.au; 4 Respiratory Unit, Lyell McEwin Hospital, Elizabeth Vale, SA 5112, Australia; 5 Centre for Cancer Biology, SA Pathology and University of South Australia, Adelaide, SA 5001, Australia

**Keywords:** airway, autophagy, biochemistry, chronic obstructive pulmonary disease, diagnostic, LC3, lymphocyte, respiratory

## Abstract

Assessing autophagy may offer insights into the pathogenesis of chronic obstructive pulmonary disease (COPD). However, measuring the dynamic aspect of autophagy is challenging, and sample manipulation can cause signal fluctuations that deviate from physiological conditions. We applied an organotypic method to quantify autophagy in COPD, where it frequently demonstrates disease-related dysregulation. Blood from control and COPD participants was treated with or without chloroquine. Microtubule-associated protein 1 light chain 3B II (LC3B-II) abundance was quantified in peripheral blood mononuclear cells (PBMCs), and findings were validated by transmission electron microscopy. Our observations show that while basal LC3B-II abundance was similar between groups (*p* = 0.60), autophagic flux was significantly lower in the COPD cohort, suggesting disruption in the regulatory factors that direct autophagosome clearance (*p* = 0.004). This was supported by less frequent observations of autophagy-related vacuoles in the cytosol of COPD-derived PBMCs. Our findings indicate that the suppression of autophagy can be detected in the blood of individuals with COPD, which warrants further investigation into its contribution to extrapulmonary disease processes.

## 1. Introduction

Chronic obstructive pulmonary disease (COPD) is among the group of conditions in which dysregulated macroautophagy (hereafter referred to as autophagy) is understood to potentiate disease and may contribute to causation [1]. Many disease phenotypes seen in COPD, including the autoimmune-like inflammation [2], persistent infection [3], and accelerated senescence [4], align with the consequences of restricted autophagic flux. Despite these findings, there remains an important debate to determine whether autophagy is restricted, and thereby diminishing its homeostatic support, or accelerated as a maladaptive response to persistent COPD-related exposures (reviewed in [5]). In broad terms, this uncertainty is because COPD is complex, has a genetic component, and is typically diagnosed at an advanced stage when the convergence of several pathophysiological events obscure causal molecular patterns. Coupled with this, assessing autophagy function in a manner that reliably reflects the situation in vivo remains challenging [6]. Hence, despite the significant promise of targeting autophagy to manage COPD, the world’s third most lethal disease [7], autophagy remains largely disconnected from clinical, diagnostic, and pharmacological inquiry.

Addressing the measurement problem, Bensalem et al. (2021) developed a laboratory method to assess autophagic flux by quantifying lipidated microtubule-associated protein 1 light chain 3B II (LC3B-II) in clinically derived human blood samples [8]. Upon lipidation, LC3B-I is converted to LC3B-II, which associates with autophagosome membranes and is widely used as a marker of autophagosome formation and autophagic flux. However, meaningful interpretation of LC3B-II requires preservation of the cellular context in which autophagy operates, as ex vivo manipulation can rapidly distort autophagic activity. This has limited the application of autophagy measurements in clinically derived samples. By treating one blood sample with chloroquine (CQ) and comparing LC3B-II accumulation to basal levels in an untreated sample, they effectively evaluated autophagic flux in peripheral blood mononuclear cells (PBMCs) from different participants [8]. The value of this method is that it assesses autophagy in an organotypic manner—taking blood in the clinic requires minimal clinical resource, the period from collection to protein isolation is brief, and the blood maintains the PBMCs as if they were still in the body. While the lethal processes ascribed to COPD occur in the airways, this condition is associated with serious systemic effects [9]. The products of inflammation and inhaled environmental factors (e.g., chemicals from tobacco and occupational/environmental exposures) are transmitted to cells of the circulatory system [10,11]. Furthermore, disease-associated genetic variants are present in all somatic cells, such that immune cells encoding COPD susceptibility alleles may exhibit intrinsic alterations in autophagy alongside exposure-driven effects. This provides a rationale for assessing autophagy in PBMCs as a systemic readout of COPD-related biology. Indeed, the pleiotropic nature of many COPD risk variants, capable of influencing both pulmonary and systemic traits such as metabolic syndrome and inflammation, is supported by genome-wide studies that often source genetic material from non-airway tissues [12]. Autophagy-related genes are linked (or are convincing candidates) to the development of human disease, including COPD, asthma, and pulmonary fibrosis [13], and we are now identifying the relationship between the peripheral consequences of COPD and dysregulation of autophagy [14,15,16]. Hence, an interesting question that arises from these findings is whether assessing autophagy in the peripheral circulation can inform the development of a non-invasive screen for COPD.

Here, we assessed autophagy in PBMCs isolated from individuals suffering from COPD to evaluate alterations in autophagic flux. Our observations show that PBMCs from participants with COPD exhibit arrested autophagy compared to the outcomes from healthy controls.

## 2. Results

### 2.1. PBMCs from COPD Participants Exhibited Reduced Autophagic Flux

**Figure 1:**
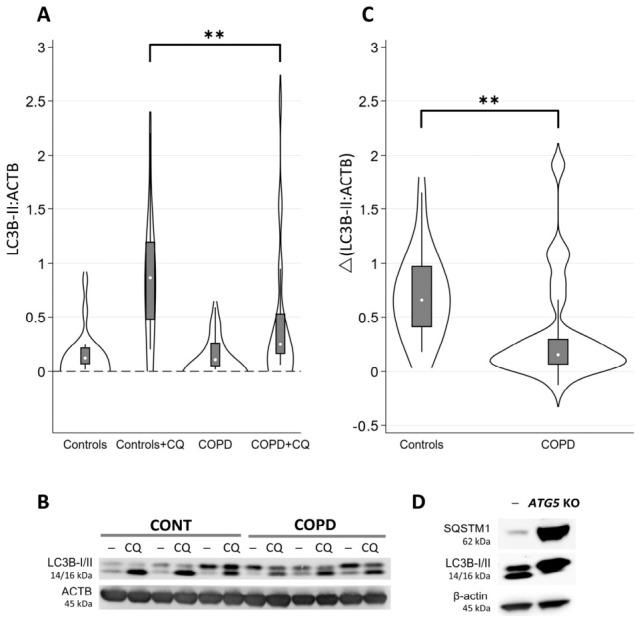
Peripheral blood mononuclear cells from individuals with COPD exhibited reduced autophagic flux. (**A**) Basal level LC3B-II observation densities show a near-zero distribution for both groups, while the COPD group exhibited a significant reduction in LC3B-II accumulation with chloroquine (CQ) vs. the controls. (**B**) A representative blot comparing no-treatment and CQ outcomes for LC3B-I/II and Actin-β (ACTB, (**C**)). Measures of autophagic flux (△LC3B-II) showed a significant reduction in autophagy competency for the COPD group. (**D**) The LC3B-I/II antibody was validated using protein isolated from 16HBE14o-cells transduced with a CRISPR *autophagy-related 5* (*ATG5*) KO construct. The depletion of ATG5 protein reduced detectable LC3B-II (the lipidated form of LC3B-I) and resulted in substantial increases in the LC3B-I and SQSTM1 signals. Outcomes for LC3B-II were normalized to the Actin-β signal. Control, n = 15 and COPD, n = 22. “**” denoted p < 0.01. Outcomes for the violin graphs were from non-parametric analyses; Wilcoxon matched-pairs sign-rank test when comparing no-treatment and CQ, and Kruskal–Wallis rank test for comparisons between control vs. COPD. Violin graph boxplots show the mean ± standard error, and whiskers denote ±95% CI.

The observations for LC3B-II protein abundance in PBMCs were first compared separately for each group/exposure (Figure 1A,B; participant demographics are shown in Table 1). PBMCs from either group exhibited a similar near-zero signal distribution for the untreated condition (*p* = 0.60, control vs. COPD), indicating low basal LC3B-I lipidation for PBMCs, which is normal for these groups. Both control and COPD cells also demonstrated an elevation in LC3B-II abundance after exposure to CQ (both *p* < 0.001, CQ vs. NT). However, control PBMCs accumulated an appreciably larger amount of LC3B-II in response to CQ over the exposure interval (*p* < 0.007, control vs. COPD), suggesting that PBMCs from COPD participants exhibit restriction in the processes contributing to autophagic flux (see also Figure S1). Values for autophagic flux (△LC3B-II) were derived from the difference between LC3B-II abundance in CQ-treated and untreated samples, reflecting LC3B-II accumulation following lysosomal inhibition. As shown in Figure 1C, the COPD group exhibited significant autophagic insufficiency when compared to the control group (*p* = 0.004, control vs. COPD). The observations for autophagic flux were further assessed to determine whether differences in age and gender were significant confounding variables (model-based analysis). As shown in Table 2, the reduction in autophagic flux determined for the COPD group remained significant after accounting for the covariates. However, estimates derived within the model show that age (gender was a non-significant confounder) can influence outcomes related to autophagic flux. The efficacy/specificity of the LC3B-I/II antibody was also assessed using lentivirus CRISPR KO directed to *autophagy-related 5* (*ATG5*). Depletion of the ATG5 protein caused a reduction in ATG5-mediated lipidation of LC3B-I (i.e., a reduction in LC3B-II), concomitant with an elevated abundance of p62/Sequestosome-1 (SQSTM1) (Figure 1D).

### 2.2. Autophagosome Abundance Is Decreased in PBMCs Exposed to CQ in COPD vs. Control

**Figure 2:**
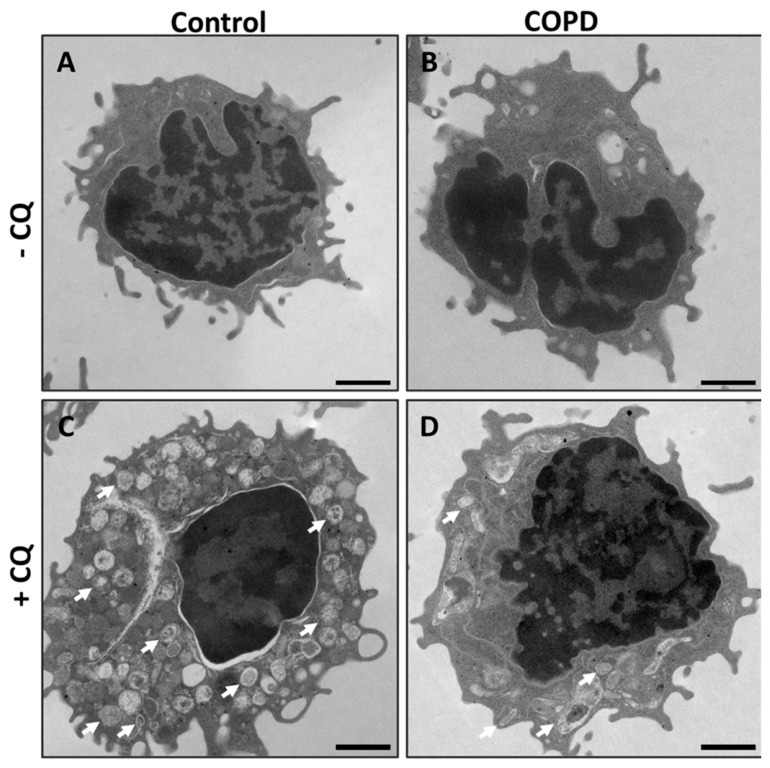
Autophagy-related vacuoles accumulate in PBMCs following chloroquine exposure. (**A**–**D**) Representative transmission electron micrographs of untreated peripheral blood mononuclear cells (PBMCs) isolated from control and COPD participants or following exposure to chloroquine (CQ; 150 μM, 1 h). (**A**,**B**) PBMCs from control and COPD participants under untreated conditions showed low basal levels of autophagy, with autophagy-related vacuoles rarely detected. (**C**,**D**) Exposure to CQ resulted in the accumulation of autophagy-related vacuoles (examples indicated by white arrows). Consistent with the biochemical flux measurements shown in Figure 1, vacuoles appear more abundant in control PBMCs compared with COPD-derived PBMCs. Scale bars: 1 µm.

High-sensitivity microscopy was performed to directly inspect PBMCs for alterations in autophagosome frequency between the control and COPD conditions. As shown in Figure 2A,B, vesicular structures indicative of autophagy-related vacuoles were rarely detected in either control or COPD-derived PBMCs. However, the accumulation of autophagy-related vacuoles increased in control PBMCs challenged with CQ. In conjunction with the outcomes from protein biochemistry, these observations are consistent with reduced autophagic flux in PBMCs obtained from individuals with COPD.

## 3. Discussion

We show that autophagic flux is reduced in PBMCs from individuals with COPD using an organotypic CQ flux assay (Figure 1). This method afforded a temporal window to assess autophagy in an organotypic manner (i.e., ex vivo exposure to CQ), which enabled us to quantify and compare the relative magnitude of autophagic function available to PBMCs from control and COPD participants. Comparative reports for COPD and related conditions that exhibit significant systemic inflammation, for the most part, explain the involvement of autophagy in disease processes with snapshots of LC3B-II and, although not always, an autophagy-receptor substrate (e.g., [17,18,19,20]). This can provide important information, but is not sufficient to describe how disease-related phenomena connect to the perturbations in autophagic competency. As flux implies a process (i.e., with a start and endpoint), it is important to consider the dynamic aspect of autophagy to provide information pertaining to function. Furthermore, and important for PBMCs, measures of autophagy receptor abundance do not always correlate with the outcomes for autophagic flux inferred by the abundance of LC3B-II (e.g., [8,21,22]). Indeed, we tested three autophagy receptor proteins (neighbor of BRCA1 gene 1, Tax1-binding protein 1, and SQSTM1) in addition to phospho-ATG16L1, which is reported to correlate with autophagy induction [23], and neither provided outcomes that were consistent with the LC3B-II observations in PBMCs (Figure S3). This issue has been observed by several others (e.g., [8] for PBMCs) and underscores the challenges that need to be considered when assessing autophagy in a meaningful way. Importantly, here we support protein biochemistry using high-power microscopy (Figure 2) to observe autophagosomes directly. Undoubtedly, these measurement complexities are central to the current debate as to whether autophagy is inappropriately attenuated or increased as a maladaptive response to COPD-related disease phenomena. Our findings, at least for PBMCs, point to a scenario whereby those COPD-related phenotypes that can be logically aligned with autophagic dysregulation arise from inappropriate restriction of autophagic flux. Collectively, these findings (Figure 1 and Figure 2) support reduced autophagic flux in COPD-derived PBMCs.

The utility and limitations of these outcomes can be addressed in terms of whether measures of autophagy can be used as a prospective diagnostic readout for COPD. While assessing blood is an attractive non-invasive tissue for diagnostic purposes, a central question is whether assessing autophagy in PBMCs is a suitable proxy for the airways. Despite our findings and the increasing evidence that peripheral lymphocytes are altered in COPD [11,24], the vast airway epithelial and macrophage interface is the first to respond to most COPD-related environmental and microbial exposures. Hence, a rational position is that the assessment of lung tissues should offer more informative predictive insights, particularly during early disease. Indeed, outcomes derived from lung samples have been convincingly shown to exhibit autophagy incompetency in COPD [3,25,26]. Further, the abundance of autophagy receptors (to support LC3B-II observations), e.g., in airway epithelial cells, generally correlate with LC3B-II levels, meaning that their analysis can provide a robust assessment of autophagic flux [3]. Efforts in our studies using airway biopsies remain limited by blood and mucin co-contamination (e.g., airway brushing biopsies), which produces significant signal deterioration. To address this, we are currently developing/optimizing a model to reassess airway samples using an adapted version of the method applied here. In terms of achieving a balance of disease/control participant demographics, our findings and observations from other groups support the position that chronological age informs (and is influenced by) the state of the biochemical processes that regulate autophagy (Table 1; [25,27,28]). This is demonstrated in COPD, where accelerated senescence and unscheduled apoptosis are hallmarks of disease and are associated with the dysregulation of autophagy [4,29,30,31]. While our evaluation of autophagy remained significant after correcting for the age disparity, assessments using homogeneous participant groups will be a central prerequisite leading to a diagnostic model that is predictive of COPD. Detailed assessment of COPD severity (including GOLD stage), pharmacologic treatment history, environmental exposures, and recent exacerbations were not performed in the current cohort and should be incorporated into future studies designed to evaluate their influence on autophagy-related outcomes. These data were not prospectively collected in a standardized manner as part of the original study design and therefore could not be reliably incorporated into the present analysis. Hence, supported by the current outcomes, we are now examining large COPD/control cohorts across three hospital sites, matching both demographic (e.g., gender) and clinical variables (e.g., COPD GOLD staging, relevant occupational, environmental, and therapeutic exposures, and recent exacerbations). One overarching limitation of this approach is the reliance on Western blotting, a method that is labor-intensive, susceptible to interexperimental variation, and is a semiquantitative assessment of protein abundance. These limitations are particularly relevant in autophagy studies, where accurate interpretation requires additional validation to distinguish between increased flux and impaired autophagosome degradation.

## 4. Methods

### 4.1. Participants

Ethics approval was granted by the Central Adelaide Local Health Network Human Research Ethics Committee (CALHN reference number: 12978). All participants provided written informed consent. Samples were obtained by the clinical team at the Royal Adelaide Hospital’s Department of Thoracic Medicine and Queen Elizabeth Hospital’s Respiratory Medicine Unit. Control participants were recruited from the same clinical services and were required to be never-smokers with no history of chronic respiratory disease. Participant demographics are shown in Table 1. Control participants reported no history of chronic respiratory disease and were never smokers. Eligibility was determined by clinician assessment and spirometry (FEV1/FVC ≥ 0.70), with participants screened to exclude chronic respiratory disease. COPD was determined by clinical observations and lung function test (FEV1/FVC ratio below 70%). Detailed clinical variables including GOLD stage, pharmacologic treatment history, recent exacerbation history, and environmental exposures were not prospectively collected in a standardized manner as part of the present study and were therefore not incorporated into the analysis.

### 4.2. PBMC Isolation

PBMCs were isolated without deviation using processes and products described by Bensalem et al. (2020) [8]. Briefly, blood collected in lithium-heparin tubes was entered into the PBMC isolation process no longer than 2 h after obtaining the sample from the clinic. Chloroquine diphosphate was added to a concentration of 150 µM and allowed to incubate for 1 h at 37 °C simultaneously with the untreated sample. PBMCs were isolated using the Ficoll gradient method.

### 4.3. Western Blot

Western blots were performed as previously described (e.g., [32]). Briefly, protein was isolated using M-PER Mammalian Protein Extraction Reagent (Thermo Fisher Scientific Inc., Waltham, MA, USA, 78501), and Halt Protease and Phosphatase Inhibitor Cocktail (Thermo Fisher Scientific Inc., 78441). Protein was quantified using the Pierce BCA Protein Assay Kit (Thermo Fisher Scientific Inc., 23225) and 10 μg electrophoresed in 4–12% Bis-Tris denaturing gels (Thermo Fisher Scientific, NP0321) using MOPS chemistry (Thermo Fisher Scientific, NP0001). When possible, a calibrator protein sample of known concentration was included to account for signal variation due to inter-experimental/membrane variation. Transfer was to 0.2 µm pore PVDF membranes (Bio-Rad Laboratories Inc., Hercules, CA, USA, 1704156), which were sectioned so target and normalization signals could be quantified from the same blot to mitigate loading inconsistencies, inter-membrane variation, and detection bias caused by multi-probing or strip/re-probing the membrane. Membranes were blocked in 5% skim milk for mouse anti-Actin-β (1:10,000, A1978; Sigma-Aldrich Co., St. Louis, MO, USA) and 5% BSA (Merck KGaA, Darmstadt, Germany, 126575) for rabbit anti-LC3B-I/II (Cell Signaling Technology, Danvers, MA, USA, 4108) and subsequently probed overnight at 4 °C with the same respective buffer/antibody combinations. Secondary incubation was 1 h at RT in 5% skim milk with mouse or rabbit IgG horseradish peroxidase-conjugated antibodies (R&D Systems, Minneapolis, MN, USA, HAF007 and HAF008, respectively). The suspension for blocking and antibody incubations was 1× Tris buffered saline (Thermo Fisher Scientific, J60877.K3) with 0.1% Tween 20 (Merck, P1379). Markers were sectioned off prior to imaging to prevent primary/secondary antibody cross-reaction/non-specific binding. Chemiluminescent signal production was performed with Amersham ECL Advanced Western Blotting Detection reagents (GE Healthcare, Chicago, IL, USA, RPN2135). Image acquisition was performed using the LAS-4000 Luminescent Image Analyzer (Fujifilm Life Sciences, Tokyo, Japan), with signal detection set to annotate blots that produced signals beyond a predetermined linear range for each target/probe. Histogram densitometry was performed using Multi Gauge software (Fujifilm Life Sciences, V3.1, Japan).

### 4.4. Generation of LentiCRISPR2-Atg5

Briefly, HEK-293T cells were co-transfected with lentivirus construct encoding *Cas9* and a sgRNA targeting exon 7 of *ATG5* (LentiCRISPRv2-*ATG5*; Addgene, Watertown, MA, USA, 99573 [33]; deposited by Edward Campbell), pMDLg/pRRE (Addgene, 12251), pMD2.G (Addgene, 12259), and pRSV-REV (Addgene, 12253) (packaging vectors deposited by Didier Trono) using Lipofectamine 3000 (ThermoFisher Scientific, L3000150). Supernatant was collected 24 and 48 h after transfection, centrifuged at 3000 rpm, filtered (Corning, Corning, NY, USA, 430768), and ultra-centrifuged (18,000 rpm; ThermoFisher Scientific, Sorvall WX80). After ultra-centrifugation, the supernatant was removed and the pellet resuspended and preserved at −80 °C. Titration was performed by transducing HEK293T cells in a 24-well plate with LentiCRISPRv2-*ATG5*, and after 72 h transduction, the cells were collected and DNA extracted/purified. DNA for cells transduced with Lenti GFP was used as a standard ladder, and qPCR was performed and normalized to the house-keeper gene to determine LentiCRISPRv2-*ATG5* titre.

### 4.5. Generation of Atg5 KO 16HBE14o- Cells

The LC3B-I/II antibody applied here is known to be sensitive and specific for LC3B-II [34]. To verify this, 16HBE14o-cells (EMD Millipore Corporation, Burlington, MA, USA, SCC150) were used to model the functional knockout of autophagy. This cell line was chosen as 16HBE14o-produced high LC3B-1I and low SQSTM1 signals during log phase growth (i.e., to allow sensitive comparison vs. outcomes for *ATG5* KO), and to avoid applying clinical samples (i.e., PBMCs) to optimization procedures. Then, 16HBE14o-cells (EMD Millipore Corporation, SCC150) were propagated according to the manufacturer’s protocol and transduced with LentiCRISPRv2-*ATG5* at a multiplicity of infection of 10 and allowed to culture for 48 h. Selective pressure on transduced cells was applied by culturing in the presence of puromycin (ThermoFisher Scientific, J67236.XF) for 15 days, after which colonies were selected, propagated, and screened. To confirm ATG5 protein knockout, Western blot analysis was performed using anti-rabbit-ATG5 (1:2000; AbCam, Cambridge, UK, Ab108327; Figure S2). Functional inhibition of autophagy was assessed by Western blot using LC3B-I/II (as above) and SQSTM1 (Cell Signaling Technology, 5114) antibodies.

### 4.6. Transmission Electron Microscopy (TEM)

PBMC isolates were pelleted (400 g) for 5 min and resuspended/fixed in 500 µL of TEM fixative (4% formaldehyde, 1% glutaraldehyde, 4% sucrose in 1× PBS; each Sigma-Aldrich). Fixed cells were processed for mounting by the staff at the University of Adelaide Microscopy suite. Sections were examined using an FEI Tecnai G2 Spirit transmission electron microscope (Thermo Scientific, Eindhoven, The Netherlands). Autophagic vacuoles were identified as vesicular structures containing heterogeneous electron-dense cytoplasmic or membranous material and bounded by a limiting membrane. Vesicles that were completely electron-lucent or displayed uniform electron density consistent with lysosomes were excluded from quantification.

### 4.7. Statistical Analysis

For each protein, Western blot densitometry results were analyzed with a random effect of test repetition and reported relative to the abundance of Actin-β. Analysis was performed in Stata v18 (StataCorp LLC, College Station, TX, USA). LC3B-II Western blot intensities were analyzed using a gamma (log link) multi-level regression model, with log(Actin-β) intensities as an offset and a robust variance–covariance. Random effects included individuals nested within blot and CQ treatment as a crossed random factor for each individual. Fixed effects included group (COPD vs. control), CQ treatment (yes vs. no), and the group × CQ interaction. Postestimation tools were used to estimate marginal means for LC3B-II normalized to Actin-β (LC3B-II:Actin-β) and the difference in these marginal means due to CQ treatment (LC3B-II:Actin-β). Age and sex were included as additional covariates, and a full factorial model was estimated. The marginal means for both COPD participants and controls were then estimated using the age/sex covariate distribution of COPD participants, i.e., the results for the controls were estimated according to the age/sex distribution of COPD participants to allow a direct comparison.

## 5. Conclusions 

We provide evidence that autophagy is suppressed in PBMCs from COPD donors. A clear understanding of the complex nexus between autophagy and COPD, particularly in a clinically relevant context, awaits the development of more efficient analytical methods yielding explicit outcome measures. Until then, a critical goal in this area of research will be to clarify whether autophagy dysregulation in the inflamed airway is a driver of COPD pathogenesis or merely a correlative phenomenon.

## Figures and Tables

**Table 1:** Participant demographics for protein biochemistry.

	n	Age (SD)	Gender (F/M)	FEV1/FVC % (SD)
Control	15	55.3 (12.7)	6/9	78.6 (5.8)
COPD	22	67.4 (6.7)	7/15	53.6 (13.5)
Total	37	62.5 (11.2)	13/24	63.8 (16.6)

FEV1, forced expiratory volume in one second. FVC, forced vital capacity.

**Table 2:** Model-based analysis of autophagic flux adjusted for age and gender.

Covariates	ΔLC3B-II:Actin-β (95% CI)		COPD vs. Controls
	COPD	Controls	*p* Value
None	0.37 (−0.01, 0.76)	0.82 (0.42, 1.23)	<0.0001
Age, Sex ^1^	0.35 (−0.03, 0.73)	0.59 (0.24, 0.94)	0.003

^1^ Estimated at the covariate distribution of COPD patients, i.e., the predicted values if controls had been age/sex matched to COPD patients.

## Data Availability

The data presented in this study are available upon request from the corresponding author.

## Supplementary Materials

The following supporting information can be downloaded at: https://www.mdpi.com/article/10.3390/ijms27125337/s1.
