# Supplementary material for: Autophagy Is Suppressed in Peripheral Blood Mononuclear Cells During Chronic Obstructive Pulmonary Disease
Source: Int J Mol Sci. 2026 Jun 13;27(12):5337. doi: 10.3390/ijms27125337 (PMC13299432; doi:10.3390/ijms27125337)
Supplement: Supplementary file 1 [file ijms-27-05337-s001.zip › ijms-4224930-supplementary.pdf]

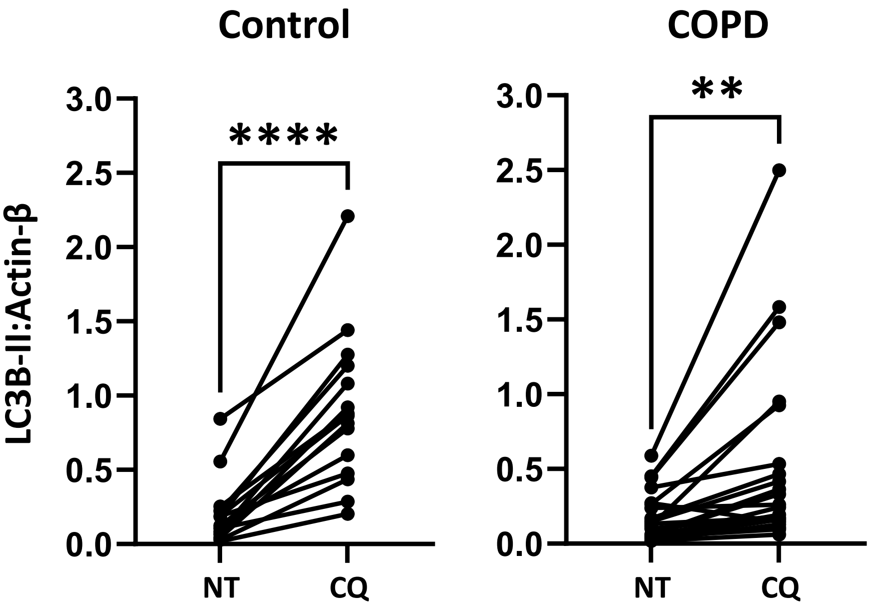

Figure S1. Chloroquine-induced elevation of LC3B-II in peripheral blood mononuclear cells is heightened in control vs COPD.

Accumulation of the LC3B-II signal is more pronounced in the control group suggesting heightened autophagosome biosynthesis in the control group during the exposure period.

*Normality was confirmed (e.g. Shapiro-Wilk and D'Agostino–Pearson tests) and analysis was performed using paired t-tests. "**" denotes P < 0.01, and "****" P < 0.0001.*

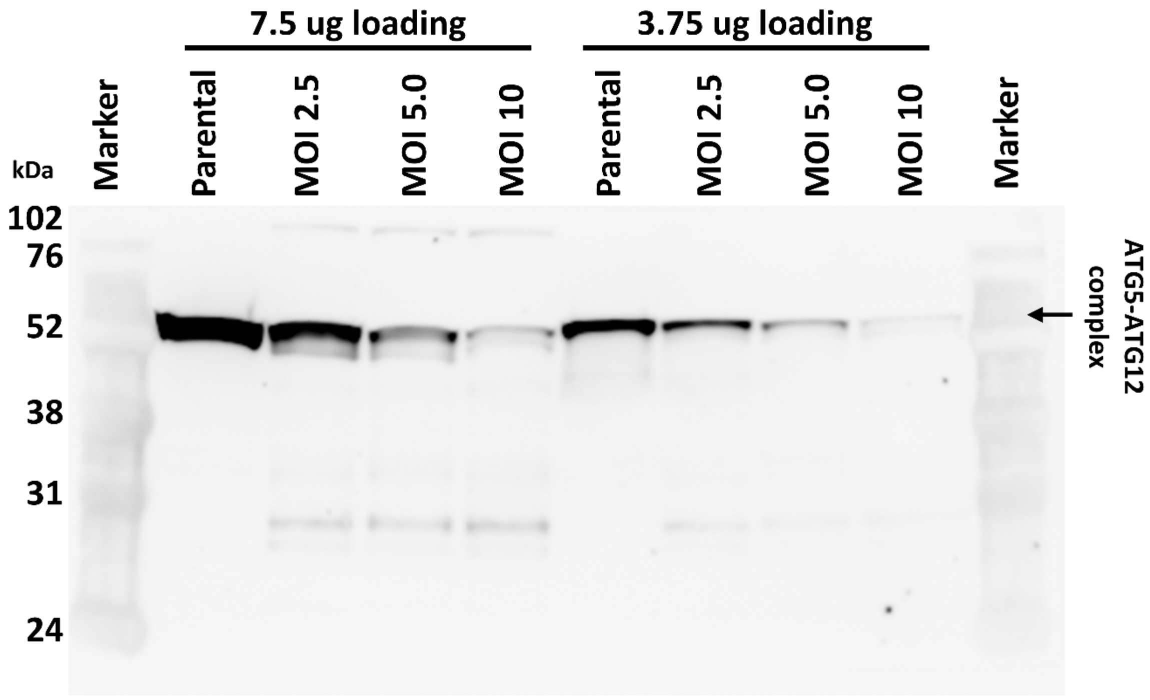

Figure S2. Validation of ATG5 knockout in 16HBE14o- cells.

Western blot analysis confirming ATG5 depletion in 16HBE14o- cells following transduction with LentiCRISPRv2-*ATG5*. Under control conditions, ATG5 is detected predominantly as the ATG5–ATG12 conjugate (~55 kDa), consistent with covalent linkage between ATG5 and ATG12. In ATG5 knockout cells, this band was lost, indicating disruption of the ATG5–ATG12 complex. Consistent with functional inhibition of autophagy, ATG5 depletion was associated with reduced LC3B-II formation and increased accumulation of SQSTM1. These findings confirm effective disruption of ATG5-dependent autophagy machinery.

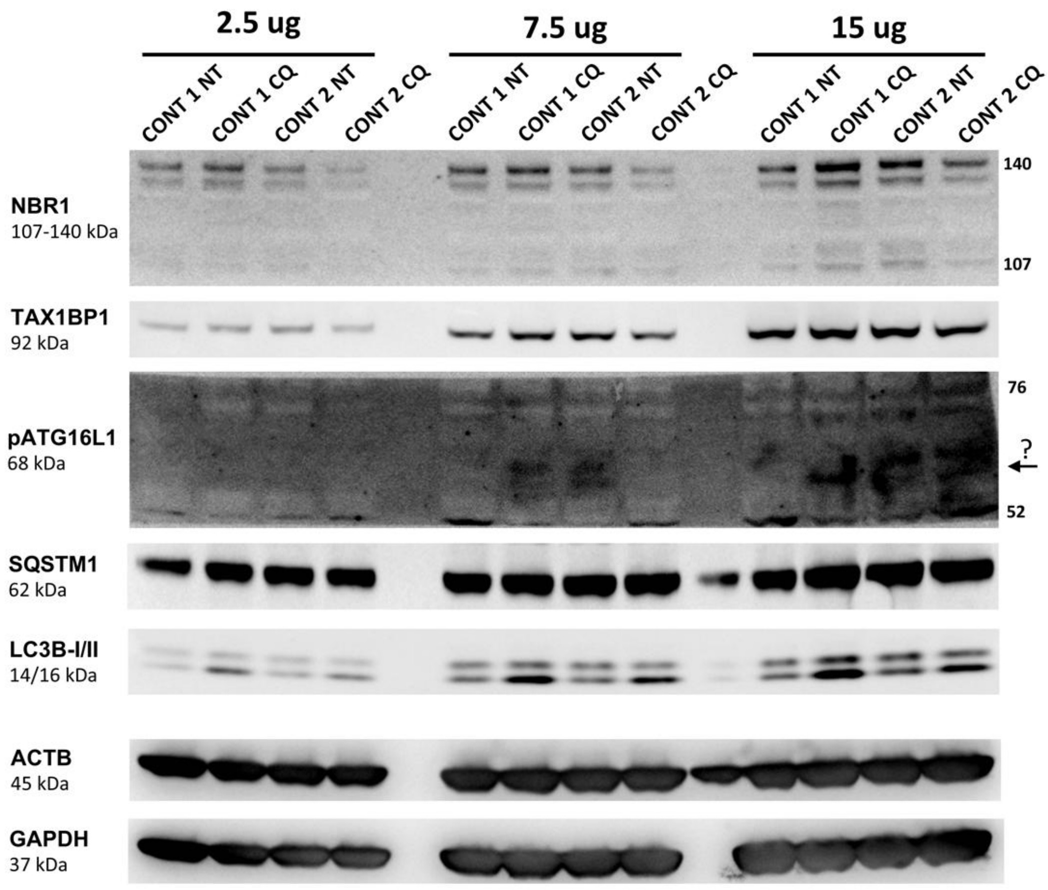

Figure S3. Autophagy receptors and pATG16L1 did not exhibit signal modulation in a manner consistent with the inhibition of autophagy in PBMCs exposed to chloroquine.

Consistently, Western blot outcomes showed the autophagy receptors Neighbor of Brca1 (NBR1), TAX1BP1 and p62/Sequestosome-1 (SQSTM1), failed to demonstrate a concomitant signal induction with LC3B-II in PBMCs exposed to chloroquine (CQ; 150 µM for 1 hour vs no-treatment control; NT). Assessment of pATG16L1 is reported to quantify autophagy flux independent of LC3 or autophagy receptor proteins, was below the detection threshold in PBMCs (normalized transcripts per million values are approximately 2.1, vs e.g. Actin-β (ACTB) with approximately 1,000 nTPM (Proteinatlas.org)). This issue was compounded by the specified blocking agent being restricted for importation into Australia (personal communication with AbCam). *Images are representative of several efforts to optimize these outcomes and are from a single membrane to mitigate inter-membrane variability and detection bias caused by multi-probing or strip/re-probing the membrane. Protein loading was 2.5, 7.5 and 15 µg to detect signal modulation that may be obscured by high signal magnitude (e.g. high protein abundance) or low signal strength (e.g. low abundance or low efficacy antibody-target interactions). Two normalizing proteins were used to account for signal modulation in either. NBR1 has been shown to migrate at either 140 or/and 107 kDa. Markers are removed to prevent primary/secondary antibody cross-reaction/non-specific binding.*
